# PDGF-R inhibition induces glioblastoma cell differentiation via DUSP1/p38^MAPK^ signalling

**DOI:** 10.1038/s41388-022-02294-x

**Published:** 2022-04-07

**Authors:** Rosemary Lane, Chiara Cilibrasi, Jianing Chen, Kalpit Shah, Eleonora Messuti, Nektarios K. Mazarakis, Justin Stebbing, Giles Critchley, Erwei Song, Thomas Simon, Georgios Giamas

**Affiliations:** 1 University of Sussex, School of Life Sciences, Department of Biochemistry and Biomedicine, Brighton, BN1 9QG UK; 2 Breast Tumor Center, Sun Yat-Sen Memorial Hospital, Sun Yat-Sen University, Guangzhou, China; 3 Genentech, Inc, South San Francisco, CA USA; 4 Royal College of Surgeons in Ireland, D02 YN77 Dublin, Ireland; 5 Department of Neurosurgery, Beaumont Hospital, D09 V2N0 Dublin, Ireland; 6 Division of Cancer, Department of Surgery and Cancer, Imperial College London, Hammersmith Hospital Campus, London, United Kingdom; 7 Department of Neurosurgery, University Hospitals Sussex, Brighton, BN2 5BE UK; 8 University of Southern California, Keck School of Medicine, Department of Translational Genomics, Los Angeles, CA 90033 USA

**Keywords:** CNS cancer, Growth factor signalling

## Abstract

Glioblastoma (GBM) is the most common and fatal primary brain tumour in adults. Considering that resistance to current therapies leads to limited response in patients, new therapeutic options are urgently needed. In recent years, differentiation therapy has been proposed as an alternative for GBM treatment, with the aim of bringing cancer cells into a post-mitotic/differentiated state, ultimately limiting tumour growth. As an integral component of cancer development and regulation of differentiation processes, kinases are potential targets of differentiation therapies. The present study describes how the screening of a panel of kinase inhibitors (KIs) identified PDGF-Rα/β inhibitor CP-673451 as a potential differentiation agent in GBM. We show that targeting PDGF-Rα/β with CP-673451 in vitro triggers outgrowth of neurite-like processes in GBM cell lines and GBM stem cells (GSCs), suggesting differentiation into neural-like cells, while reducing proliferation and invasion in 3D hyaluronic acid hydrogels. In addition, we report that treatment with CP-673451 improves the anti-tumour effects of temozolomide in vivo using a subcutaneous xenograft mouse model. RNA sequencing and follow-up proteomic analysis revealed that upregulation of phosphatase DUSP1 and consecutive downregulation of phosphorylated-p38^MAPK^ can underlie the pro-differentiation effect of CP-673451 on GBM cells. Overall, the present study identifies a potential novel therapeutic option that could benefit GBM patients in the future, through differentiation of residual GSCs post-surgery, with the aim to limit recurrence and improve quality of life.

## Introduction

Glioblastoma (GBM) is the most common and fatal primary brain tumour in adults [1, 2] with a median survival time of 14.6 months and 5 year survival rates of less than 5%, with rates decreasing with advanced age [3]. The addition of alkylating agent temozolomide (TMZ) to the treatment regimen following maximal safe surgical resection and radiotherapy only modestly improves patients’ median survival from 12.1 months to 14.6 months [4]. Further advancements in treatment options are lacking despite a large number of studies focussed on this disease [5–7].

While numerous therapeutic approaches aim for cancer cell death, a few studies have instead started exploring treatments that induce cancer cell differentiation into non-neoplastic cells as a novel strategy [5]. Indeed, differentiation therapy has been shown to be successful in acute promyelocytic leukaemia (APL) when leukaemic promyelocytes differentiated into mature granulocytes upon treatment with the vitamin A derivative all-trans-retinoic acid (ATRA) [8]. Similar differentiation strategies have therefore been tested on other cancer types. A combination of the MEK inhibitor PD98059 and anti-diabetic drug Rosiglitazone have been reported to induce the differentiation of breast cancer cells into functional adipocytes, hence reducing metastasis in vivo [9]. Treatment of prostate cancer cell line PC-3 with inosine monophosphate (IMP) dehydrogenase inhibitors has been described to trigger maturation of cancer cells into normal prostate luminal cells in an intermediate state of differentiation [10].

GBM treatment could highly benefit from differentiation therapies. High resistance to classic cytotoxic treatments and the presence of pluripotent cancer stem cells (CSCs) in residual tumour tissues due to incomplete surgical excision make GBM a good candidate for differentiation therapies [11–16]. Several potential molecular targets of interest have thus been tested and reported for cancer cell differentiation therapy in GBM, including activators of cyclic adenosine monophosphate (cAMP), which reverse the Warburg effect and cause a metabolic shift that drives differentiation of tumour cells into astroglia [17]. Further studies reported that in neural stem cells dopamine receptor D4 (DRD4) antagonists inhibit GBM growth, induce G0/G1 arrest and promote tumour cell differentiation and apoptosis in vitro [18]. In addition, as kinases are involved in the development and progression of cancer, including GBM, and due to their activity in regulating differentiation, recent reports have suggested targeting kinases in differentiation therapies against GBM [19–21]. Kinase inhibitors (KIs) with the potential to induce differentiation in GBM include aurora-A inhibitors alisertib and MLN8237, which were able to induce senescence, differentiation, and cytotoxicity in a GBM neurosphere model and GBM cells in vitro, respectively [22, 23].

In accordance with these reports, the present study describes the screening of a panel of KIs that led to the identification of a receptor tyrosine kinase (RTK) inhibitor, namely CP-673451, which targets the platelet-derived growth factor receptor α/β (PDGF-Rα/β) as a potential differentiation agent in GBM. Herein, we propose that CP-673451 treatment can induce the upregulation of phosphatase dual-specificity phosphatase 1 (DUSP1) and consecutive downregulation of phosphorylated p38 mitogen-activated protein kinase (MAPK), leading to the onset of differentiation of GBM cells, thus limiting proliferation and invasion in vitro, while improving the anti-tumour effects of temozolomide in vivo.

## Results

### Kinase inhibitor screening identifies agents that induce neurite-like process outgrowth in U87 GBM cells

Forty-seven KIs were selected from the KI library (SelleckChem) on the basis of high potency with low IC_50_, no previously identified undesirable effects (i.e., apoptosis) at the required concentrations and with the ability to cross the blood-brain barrier (BBB) [24–27]. A full list of the selected KIs, their respective targets and significance of effect on neurite-like process outgrowth (*p*-value) can be found in Table 1.

**Table 1:** List of KIs included in the screening, their respective molecular targets and *p*-value of average length of neurite-like process per cell.

Kinase inhibitor	Target	*p*-value (average length of branching)	Kinase inhibitor	Target	*p*-value (average length of branching)
GDC-0068	Akt	NS	BMS-265246	CDK1/2	≤0.01
Phenformin HCl	AMPK	NS	CHIR-124	Chk1	≤0.01
LY294002	PI3Kα/δ/β	NS	10058-F4	c-Myc	NS
LY2835219	CDK4/6	≤0.001	HMN-214	PLK1	≤0.01
IM-12	GSK-3	NS	GSK461364	PLK1	NS
Ridaforolimus (MK-8669)	mTOR	≤0.05	NSC 23766	RAC	NS
OSU-03012 (AR-12)	PDK-1	NS	Nilotinib (AMN-107)	BCR-ABL	NS
BX-912	PDK-1	NS	CNX-774	BTK	≤0.05
ZSTK474	Class I PI3Ks	NS	TAE226 (NVP-TAE226)	FAK	≤0.05
BGT226 (NVP-BGT226)	Class I PI3Ks, mTOR	NS	SSR128129E	FGFR1	NS
Pelitinib (EKB-569)	EGFR	≤0.001	KX2-391	SRC	≤0.01
OSI-420	EGFR	NS	JNK-IN-8	JNK1/2/4	NS
AZD9291	EGFR	≤0.05	PD184352 (CI-1040)	MEK1/2	≤0.05
Chrysophanic Acid	mTOR, EGFR	NS	Trametinib (GSK1120212)	MEK1/2	≤0.05
CP-673451	PDGFRα/β	≤0.001	LY2228820	p38 MAPK	NS
Masitinib (AB1010)	PDGFR, c-Kit	NS	ZM 336372	C-RAF	NS
Tie2 kinase inhibitor	Tie-2	NS	TAK-632	RAF	NS
Vandetanib (ZD6474)	VEGFR2	≤0.05	CEP-33779	JAK2	NS
Semaxanib (SU5416)	VEGFR (FLK1/KDR)	NS	Pacritinib (SB1518)	JAK2, FLT3	≤0.001
Sunitinib Malate	VEGFR, PDGFRβ, c-Kit	NS	SMI-4a	PIM1	NS
NU7441 (KU-57788)	DNA-PK, PI3K	≤0.01	GNE-7915	LRRK2	NS
MLN8054	Aurora A	NS	PFK15	PFKFB3	NS
Aurora A Inhibitor I	Aurora A	≤0.05	KN-62	CaMKII	NS
BS-181 HCl	CDK7	NS			

**Fig. 1:**
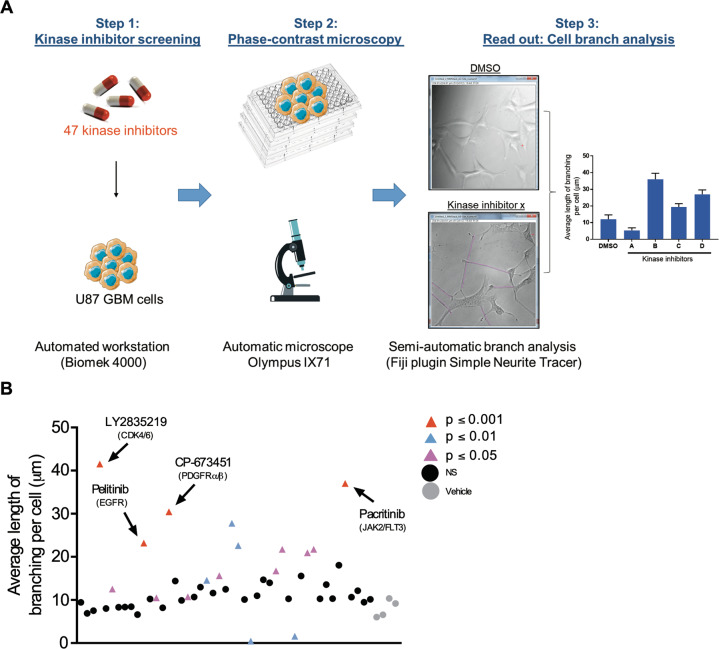
Kinase inhibitor screening identifies agents that induce neurite-like process outgrowth in U87 GBM cells. **A** Step 1: U87 GBM cells were seeded (2 × 10^3^ per well) into 96-well plates using the Biomek 4000 automatic workstation. Following incubation for 24 h, cells were treated with 1 μM of the selected KIs (*n* = 47). Step 2: After 24 h phase-contrast microscopy images were taken at 20x mag using the Olympus IX71 microscope (Micromanager software). Step 3: The length of long thin extensions termed ‘neurite-like process’ were analysed, using the Fiji plugin ‘simple neurite tracer’ (Image J) and the average length neurite-like process per cell determined. **B** U87 GBM cells were treated for 24 h with 1 μM 47 kinase inhibitors. The average length of neurite-like process was measured and normalised by cell number. The mean ± SD of *n* = 3 independent experiments is shown. Grey circles vehicle control, black circles not significant (NS) *p* ≥ 0.05, pink triangles *p* ≤ 0.05, blue triangles *p* ≤ 0.01, red triangles *p* ≤ 0.001 (two-tailed *t*-test). Kinase inhibitor ‘hits’ from the screening *p* ≤ 0.001 are indicated with black arrows and include; LY2835219 (CDK4/6), pelitinib (EGFR), CP-673451 (PDGF-Rα/β) and pacritinib (JAK2/FLT3).

Neurite-like process outgrowth was quantified following treatment to select KIs with potential differentiating effects. A schematic diagram illustrates the experimental design (Fig. 1A) with U87 GBM cells treated with 1 μM of each KI (*n* = 47) for 24 h before nine images were taken per well. All images were analysed for total neurite-like process length and normalised by cell number to obtain an average neurite-like process length per cell (Fig. 1B). The grey circles indicate the vehicle control DMSO. Black circles indicate KIs with no significant effect on neurite-like process outgrowth as compared to the control (*p* ≥ 0.05), pink, blue and red triangles indicate KIs with significant effects compared to the control (*p* ≤ 0.05, *p* ≤ 0.01 and *p* ≤ 0.001, respectively). Inhibitors showing effects with *p* ≤ 0.001 were selected for further experiments, namely: LY2835219, a potent and selective inhibitor of CDK4/6 [28], pelitinib, a potent irreversible epidermal growth factor receptor (EGFR) inhibitor [29], CP-673451, a selective inhibitor of PDGF-Rα/β [26] and pacritinib a selective dual inhibitor of Janus kinase 2 (JAK2) and FMS-like tyrosine kinase-3 (FLT3) [30] (Fig. 1B).

### Selected kinase inhibitors induce neurite-like process outgrowth in three different GBM cells and astrocytes

**Fig. 2:**
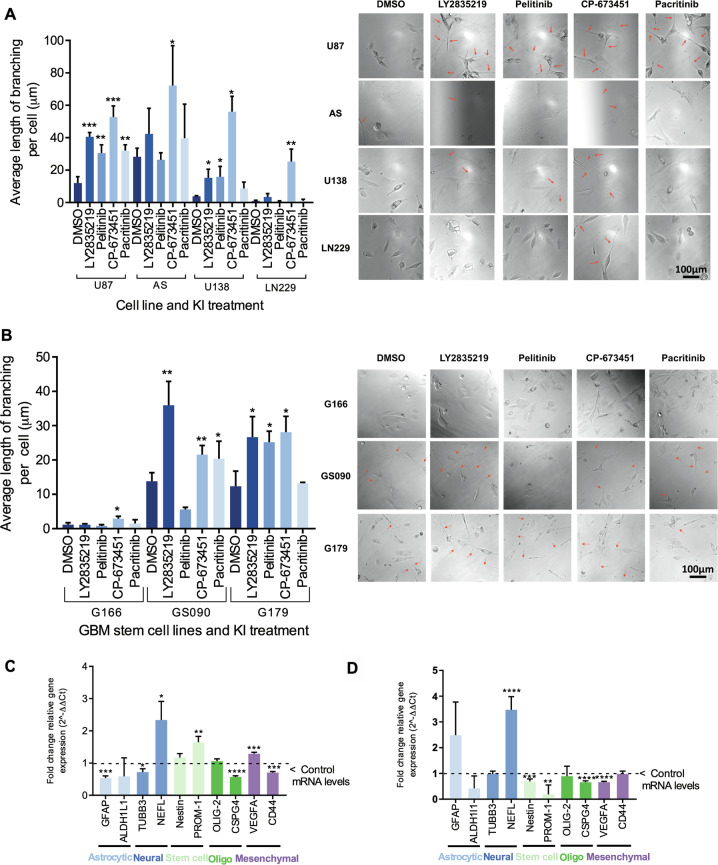
Selected kinase inhibitors induce neurite-like process outgrowth in GBM cells, GSCs and astrocytes. **A** Average length of neurite-like process per cell was determined for normal AS and GBM cell lines U87, U138 and LN229 treated for 24 h with 1 μM of the kinase inhibitor hits from the screening (LY2835219 (CDK4/6), pelitinib (EGFR), CP-673451 (PDGF-Rα/β) and pacritinib (JAK2/FLT3)). Representative images shown, red arrows indicating neurite-like processes. Images taken at 20x mag, Scale bar = 100 μm. **B** Average length of neurite-like process per cell was determined for patient-derived GSCs G166, GS090 and G179 treated for 48 h with 1 μM of the kinase inhibitor hits from the screening (LY2835219 (CDK4/6), pelitinib (EGFR), CP-673451 (PDGF-Rα/β) and pacritinib (JAK2/FLT3)). Representative images shown, red arrows indicating neurite-like processes. Images taken at 20x mag, Scale bar = 100 μM. **C**, **D** qRT-PCR performed on U87 GBM cells (**C**) and G179 GSCs (**D**) treated with 1 μM CP-673451 for 96 and 48 h, respectively, measuring the relative mRNA expression levels (fold change normalised to GAPDH compared to control levels) of cell specific markers astrocytic (GFAP, ALDH1L1), neural (β3-tubulin, NEFL) stem cell (Nestin, PROM-1) oligodendrocyte (Oligo) (OLIG-2, CSPG4) and mesenchymal (VEGFA, CD44). Average of a minimum three biological repeats *t*-test *p ≤ 0.05, **p ≤ 0.01, ***p ≤ 0.001 and ****p ≤ 0.0001.

The effects of the selected inhibitors were further assessed on a panel of GBM cell lines including U87, U138 and LN229 and astrocytes (AS), as a normal brain cell type control [31]. Neurite-like process outgrowth was measured following 24 h treatment with the inhibitors. Nine images were taken per well and the average neurite-like process length per cell was calculated as before (Fig. 2A). All four KIs identified in the screening, LY2835219, pelitinib, CP-673451 and pacritinib caused a significant increase in average neurite-like process length compared to control (12.1 ± 3.9 μm) in U87 GBM cells: 40.5 ± 2.7 μm (*p* ≤ 0.001), 30.5 ± 5.1 μm (*p* ≤ 0.01), 52.8 ± 6.9 μm (*p* ≤ 0.001) and 32.1 ± 3.6 μm (*p* ≤ 0.01), respectively. In AS, a significant increase of the average neurite-like process length per cell was observed following treatment with CP-673451 (72.1 ± 24.63 μm (*p* ≤ 0.05)) as compared to the control (28.2 ± 5.3 μm). CP-673451 similarly caused the average neurite-like process length per cell to increase significantly in LN229 cells (25.3 ± 7.7 μm (*p* ≤ 0.01)) as compared to the control (0.9 ± 0.5 μm). Finally, LY2835219, pelitinib and CP-673451 treatments caused a significant increase in the average neurite-like process length (15.3 ± 5.2 μm (*p* ≤ 0.05), 15.9 ± 6.4 μm (*p* ≤ 0.05) and 56.0 ± 9.6 (*p* ≤ 0.001) respectively) in the U138 cells compared to the control (3.9 ± 0.3 μm) (Fig. 2A).

To examine how various treatments affected cell viability and growth, metabolic activity and proliferation were measured following treatment with the inhibitors of interest using WST-1 (Supplementary Fig. 1A) and crystal violet assays (Supplementary Fig. 1B), respectively. An increase of viability was observed in U138 cells following treatment with LY2835219 1.06 ± 0.24 RAU (*p* ≤ 0.01) compared to the control, while a slight decrease in viability was observed in U138 and LN229 GBM cells following treatment with pacritinib (0.9 ± 0.05 RAU (*p* ≤ 0.05) and 0.8 ± 0.06 RAU (*p* ≤ 0.05), respectively). Treatments with LY2835219, pelitinib and pacritinib were also observed to significantly affect LN229 cell proliferation compared to the control (0.8 ± 0.09 RAU (*p* ≤ 0.01), 0.8 ± 0.1 RAU (*p* ≤ 0.05) 0.8 ± 0.1 RAU (*p* ≤ 0.05), respectively). Altogether, treatment with 1 μM of the selected KIs appeared to stimulate neurite-like process outgrowth in most studied GBM cell lines and astrocytes, with limited impact on cell viability and proliferation.

### Selected kinase inhibitors induce neurite-like process outgrowth in patient-derived GSCs

As one of the drivers of GBM recurrence with multipotent abilities, GSCs are the putative main targets of differentiation therapy. For these reasons, the selected KIs were used to treat different GSC lines, including G166, GS090 and G179. Their morphology was observed using light microscopy following treatment and the average neurite-like process length per cell was calculated as before (Fig. 2B). Treatment with 1 µM CP-673451 caused a significant increase in the average neurite-like process length (2.9 ± 0.7 μm (*p* ≤ 0.05)) in G166 cells as compared to control (1.2 ± 0.6 μm). In addition, 1 µM LY2835219, CP-673451 and pacritinib led to significant increases in the average neurite-like process length (35.9 ± 7.0 μm (*p* ≤ 0.01), 21.6 ± 2.7 μm (*p* ≤ 0.01) and 20.3 ± 5.1 μm (*p* ≤ 0.05), respectively) in GS090 GSCs compared to the control (13.8 ± 2.5 μm). Finally, 1 µM LY2835219, pelitinib and CP-673451 significantly increased the average neurite-like process length per cell (26.7 ± 6.0 μm (*p* ≤ 0.05) 25.2 ± 3.2 μm (*p* ≤ 0.05) and 28.1 ± 4.6 μm (*p* ≤ 0.05), respectively) in G179 GSCs, compared to the control (12.3 ± 4.4 μm). Representative light microscopic images are shown, with neurite-like processes indicated by red arrows.

GSC viability (WST-1) (Supplementary Fig. 1C) and proliferation (crystal violet) (Supplementary Fig. 1D) were also assessed following treatment with each of the selected KIs. The viability assay indicated a significant decrease in viability in G166 as compared to the control when treated with LY2835219 and pelitinib (0.8 ± 0.09 RAU (*p* ≤ 0.05) and 0.9 ± 0.07 RAU (*p* ≤ 0.05), respectively). On the other hand, cell viability increased significantly following treatment with CP-673451 (1.04 ± 0.02 RAU (*p* ≤ 0.05)) as compared to the control. Further, proliferation assays indicated that G166 cell proliferation decreased after pelitinib treatment (0.9 ± 0.04 RAU (*p* ≤ 0.01)) but increased upon treatment with CP-673451 (1.3 ± 0.14 RAU (*p* ≤ 0.05)) as compared to the control. Regarding GS090 cells, CP-673451 treatment decreased proliferation (0.99 ± 0.01 RAU (*p* ≤ 0.01)) as compared to the control. None of the KI treatments caused significant changes in G179 proliferation.

Taken together, the present data on both GBM cell lines and GSCs revealed that treatment with 1 μM CP-673451 increased the average neurite-like process length in the different cell lines and stem cells studied, with limited impact on cell viability and proliferation. These results suggest that CP-673451 treatment could induce differentiation in GBM cells and, therefore, CP-673451 was chosen as the focus of the present investigation.

The expression of phosphorylated (phos)-PDGF-Rα/β (tyr849/tyr857) was measured as well as PDGF-Rβ upon treatment with CP-673451. U87 GBM cells were treated with 1 µM CP-673451 for 0, 15 min, 1, 4, 24 and 48 h (Supplementary Fig. 2A, B) and phosphorylated levels of PDGF-Rα/β and PDGF-Rβ were measured by western blotting. The addition of CP-673451 caused a decrease in phos-PDGF-Rα/β from 15 min, followed by an increase at 1 and 4 h and decreases again at 24 and 48 h. Total PDGF-Rβ appeared to decrease following treatments at 24 and 48 h.

Gene expression of specific differentiation markers was quantified using qRT-PCR in order to determine which specific normal brain cell lineage (astrocyte, oligodendrocyte or neuronal) the GBM cells were differentiating into upon CP-673451 treatment. The selected differentiation markers were as follows; Astrocytic (GFAP and ALDH1L1), Neuronal (β3-tubulin and NEFL), stem cell (nestin and PROM-1 (CD133), oligodendrocyte (OLIG2 and CSPG4) and mesenchymal (VEGFA and CD44). Upon treatment of U87 GBM cells with 1 µM CP-673451 for 96 h (Fig. 2C, D), cells showed significant increases in NEFL (2.34 ± 0.58 fc (*p* ≤ 0.05)), PROM-1 (1.64 ± 0.18 fc (*p* ≤ 0.01)) and VEGFA (1.29 ± 0.05 fc (*p* ≤ 0.001)). Treatment also indicated significant decreases in GFAP (0.54 ± 0.07 fc (*p* ≤ 0.001)), CSPG4 (0.57 ± 0.04 fc (*p* ≤ 0.0001)) and CD44 (0.7 ± 0.03 fc (*p* ≤ 0.001)). Upon treatment with 1 μM CP-673451, G179 GSCs also showed a significant increase in the expression of NEFL (3.47 ± 0.51 fc (*p* ≤ 0.0001) and significant decreases in the expression of nestin and PROM1 (0.69 ± 0.09 fc (*p* ≤ 0.001) and 0.18 ± 0.37 fc (*p* ≤ 0.01), respectively), CSPG4 (0.65 ± 0.06 fc (*p* ≤ 0.001) and VEGFA (0.66 ± 0.03 fc (*p* ≤ 0.001).

Altogether our data showed that treatment with CP-673451 could trigger a neuron-like differentiation in GBM cells, as supported by the formation of neurite-like processes and expression of the neuronal NEFL marker in GBM cells, with concomitant decrease of expression of markers for stem cells and other brain cell types.

### Treatment with CP-673451 reduces proliferation and invasiveness of GBM cells and patient-derived GSCs

**Fig. 3:**
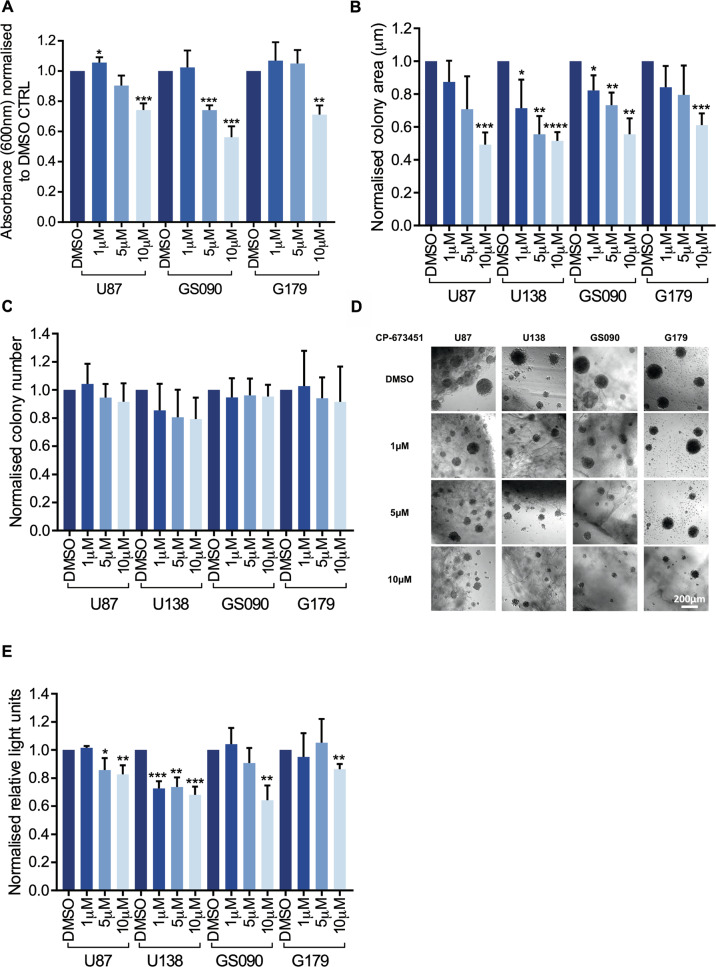
Treatment with CP-673451 reduces proliferation and invasiveness of GBM cells and patient-derived GSCs. **A** Proliferation assay (crystal violet) was performed on U87 GBM cells and patient-derived GSCs GS090 and G179 treated with increasing concentrations of CP-673451 (0, 1, 5 and 10 μM). Values normalised to DMSO control. **B** HA-based 3D assay was performed on U87, U138, GS090 and G179. Cells were seeded into the HA-based assay and incubated for 48 h before beginning CP-673451 treatment at increasing concentrations (0, 1, 5 and 10 µM). Fresh treatment was applied every 2 days. Images were taken at 10x mag on day 7 of treatment and the (**B**) areas and (**C**) numbers of colonies were measured and normalised to DMSO control. **D** Representative images are shown, Scale bar = 200 μm. **E** Viability assay (CellTiter-Glo) was then performed. Values normalised to DMSO control. The mean ± SD of minimum *n* = 3 independent experiments is displayed, representative images shown *p ≤ 0.05, **p ≤ 0.01, ***p ≤ 0.001 and ****p ≤ 0.0001 (two-tailed *t*-test).

To further characterise the effect of CP-673451 on proliferation, additional experiments with increasing concentrations of the inhibitor were performed. The U87 GBM cell line and GS090 and G179 GSCs were treated with increasing concentrations of CP-673451 (0, 1, 5 and 10 μM) for 48 h, while a proliferation assay (crystal violet) was also performed (Fig. 3A). A significant increase in U87 GBM cell proliferation was observed following treatment with 1 µM CP-673451 when compared to the control (1.1 ± 0.03 RAU (*p* ≤ 0.05)). Conversely, a significant decrease in cell proliferation was observed in GS090 cells upon treatment with 5 µM CP-673451 (0.7 ± 0.03 RAU (*p* ≤ 0.01)), as compared to the control. All three cell lines showed a significant decrease in proliferation when treated with 10 µM CP-673451: U87 (0.7 ± 0.04 RAU (*p* ≤ 0.001)), GS090 (0.6 ± 0.07 RAU (*p* ≤ 0.001)) and G179 (0.7 ± 0.06 RAU (*p* ≤ 0.01)), as compared to the control.

A HA based hydrogel assay was implemented to assess the invasiveness and colony forming ability of the U87 and U138 GBM cell lines and GS090 and G179 GSCs in a 3D model when treated with increasing concentrations of CP-673451 (0, 1, 5 and 10 µM). Cell metabolic activity was also measured in parallel (using a CellTiter-Glo luminescent cell viability assay). Both the colony areas (Fig. 3B) and colony numbers (Fig. 3C) were measured with representative images shown (Fig. 3D). A gradual decrease of the average areas of U87 and G179 cell colonies was observed, with a significant difference when compared to control upon a 10 µM CP-673451 treatment (U87: 0.5 ± 0.07 (*p* ≤ 0.001) and G179: 0.6 ± 0.07 (*p* ≤ 0.001). Following CP-673451 treatment, significant decreases in cell colony size were observed at 1, 5 and 10 µM in U138 (0.7 ± 0.17 (*p* ≤ 0.05), 0.6 ± 0.11 (*p* ≤ 0.01), 0.5 ± 0.05 (*p* ≤ 0.0001)) and GS090 (0.8 ± 0.09 (*p* ≤ 0.05) 0.7 ± 0.08 (*p* ≤ 0.01) and 0.5 ± 0.1 (*p* ≤ 0.01)). No significant alterations of the number of colonies were observed in the tested cell lines upon CP-673451 treatment. Yet, a significant decrease in U87 cell metabolic activity (Fig. 3E) was measured following CP-673451 treatment at both 5 and 10 µM (0.9 ± 0.09 (*p* ≤ 0.05) and 0.8 ± 0.06 (*p* ≤ 0.01), respectively). Similarly, U138 showed a significant decrease in metabolic activity upon treatment with 1, 5 and 10 μM CP-673451 (0.7 ± 0.05 (*p* ≤ 0.001), 0.7 ± 0.07 (*p* ≤ 0.01), 0.7 ± 0.06 (*p* ≤ 0.001)), while both GS090 and G179 GSCs showed a significant decrease in metabolic activity upon CP-673451 treatment at 10 µM (0.6 ± 0.11 (*p* ≤ 0.01)) and (0.9 ± 0.04 (*p* ≤ 0.01)), respectively. Altogether, it appears that treatment with CP-673451 limits the proliferation and invasiveness of GBM cells while stimulating their neuron-like differentiation in vitro.

### PDGF-Rα/β gene silencing induces neurite-like process outgrowth in U87 GBM cells and G179 GSCs

In the interest of ensuring the effects from pharmacological inhibition in U87 GBM cells upon CP-673451 treatment were due to specific inhibition of its targets PDGF-Rα and PDGF-Rβ, gene silencing using siRNA was performed.

PDGF-Rα and PDGF-Rβ gene expression levels were measured upon silencing. U87 GBM cells and G179 GSCs were treated with 50 nM and 100 nM control siRNA or PDGF-Rα and β siRNA combination for 48 h. Levels were normalised to control siRNA and shown as fold change (Supplementary Fig. 3A). Knockdown (KD) of PDGF-Rα could be observed in U87 GBM cells at both 50 nM and 100 nM with a reduction of 91% 0.09 ± 0.005 (*p* ≤ 0.0001) and 95% 0.05 ± 0.03 (*p* ≤ 0.0001), respectively. In addition, PDGF-Rβ expression was also reduced at both 50 nM and 100 nM by 47% 0.53 ± 0.18 (*p* ≤ 0.01) and 46% 0.53 ± 0.23 (*p* ≤ 0.05), respectively. KD of PDGF-Rα and *β* was also validated in G179 cells (Supplementary Fig. 3B). 50 nM siRNA reduced the gene expression of PDGF-Rα by 45% (0.55 ± 0.2 (*p* ≤ 0.01)) and PDGF-Rβ 48% (0.52 ± 0.2 (*p* ≤ 0.01)) compared to the control. At 100 nM siRNA, the gene expression of PDGF-Rα was reduced by 57% (0.43 ± 0.1 (*p* ≤ 0.0001)) while gene expression of PDGF-Rβ was reduced by 48% (0.52 ± 0.02 (*p* ≤ 0.001)). Further validating the siRNA transfection, the protein expression of PDGF-Rα and PDGF-Rβ was decreased in U87 GBM cells and G179 GSCs. PDGF-Rβ protein expression was reduced by 72% in U87 GBM cells at both 50 nM and 100 nM concentrations of siRNA compared to control (0.28 ± 0.1 (*p* ≤ 0.0001)) (Supplementary Fig. 3C). In G179 GSCs, 50 nM siRNA reduced the protein expression of PDGF-Rα by 37% 0.62 ± 0.1 (*p* ≤ 0.05) and PDGF-Rβ by 33% 0.67 ± 0.2 (*p* ≤ 0.05). 100 nM siRNA reduced PDGF-Rα protein expression by 26% 0.74 ± 0.1 (*p* ≤ 0.05) and PDGF-Rβ by 40% 0.60 ± 0.2 (*p* ≤ 0.05) (Supplementary Fig. 3D).

**Fig. 4:**
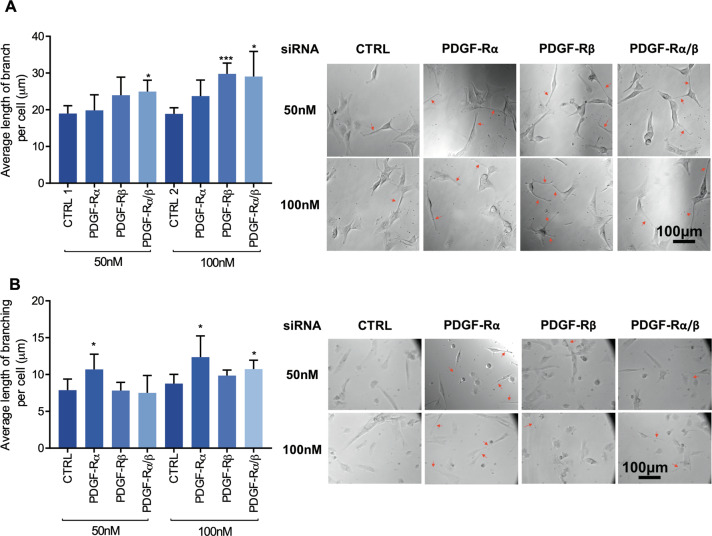
Gene silencing of PDGF-Rα/β with siRNA induces neurite-like process outgrowth in U87 GBM cells and G179 GSCs. **A**, **B** Average length of neurite-like process was determined in U87 GBM cells (**A**) and G179 GSCs (**B**) treated for 48 h with 50 nM and 100 nM siRNA CTRL, PDGF-Rα, PDGF-Rβ and PDGF-Rα and β. Representative images are shown with neurite-like processes indicated with red arrows. The mean ± SD of minimum *n* = 3 independent experiments is displayed, representative images shown *p ≤ 0.05 **p ≤ 0.01 ***p ≤ 0.001 (two-tailed *t*-test).

The morphology of the cells was observed using light microscopy and the total lengths of neurite-like processes were measured and normalised by cell number to obtain the average neurite-like process length per cell (Fig. 4A). At 50 nM compared to the control siRNA (19.0 ± 2.1 µm) only the combination of PDGF-Rα and β siRNAs increased the average neurite-like process length significantly (25.0 ± 3.1 µm (*p* ≤ 0.05)). Treatment of U87 GBM cells with 100 nM PDGF-Rβ siRNA compared to the control (18.9 ± 1.7 µm) displayed a significant increase in neurite-like process length average per cell (29.8 ± 2.9 µm (*p* ≤ 0.001)), as did the combination of PDGF-Rα and *β* siRNA (29.1 ± 6.8 µm (*p* ≤ 0.05)). Similarly, KD PDGF-Rα in G179 cells with siRNA at 50 and 100 nM significantly increased the average length of neurite-like process per cell (10.7 µm ± 2.1 (*p* ≤ 0.05)) compared to the control (7.9 ± 1.5 µm) and (12.4 ± 2.9 µm (*p* < 0.05)) compared to the control (8.8 ± 1.3 µm), respectively. KD of both PDGF-Rα and *β* also increased neurite-like process outgrowth compared to the controls (10.7 ± 1.2 µm (*p* ≤ 0.05)) (Fig. 4B).

As specific gene silencing of PDGF-Rα and PDGF-Rβ enhanced neurite-like process growth in U87 GBM cells and G179 GSCs, these data suggest that the observed impact of CP-673451 treatment on GBM cells should be, at least partly, due to specific PDGF-Rα/β inhibition.

### Combination with CP-673451 improved anti-tumour effect of temozolomide in a subcutaneous GBM mouse model

**Fig. 5:**
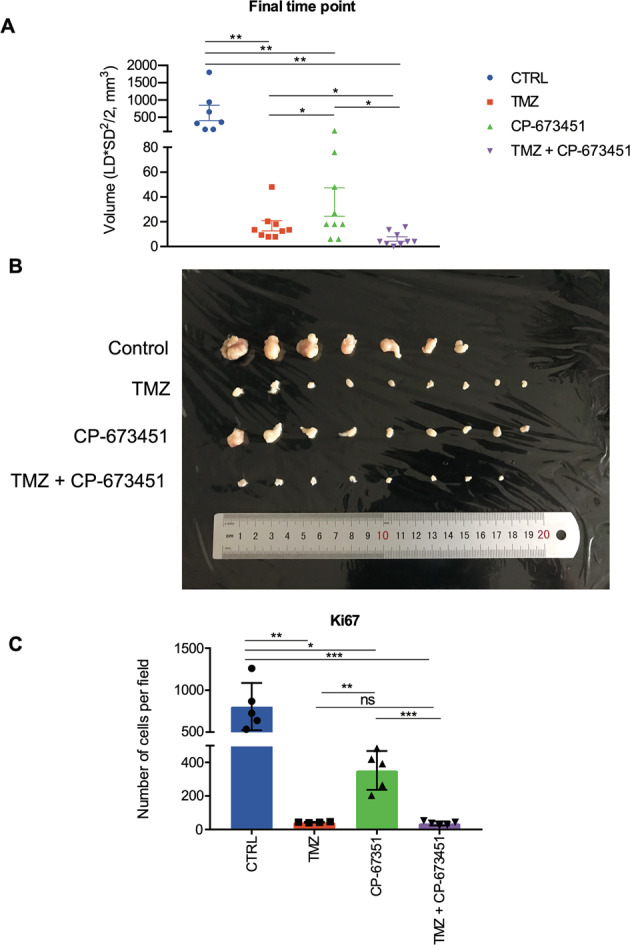
Combination with CP-673451 improved anti-tumour effect of temozolomide in a subcutaneous GBM mouse model. **A** Volumes of U87 GBM cell subcutaneous xenograft tumours from athymic nude mice were measured after 3 weeks of treatment with TMZ (25 mg/kg), CP-673451 (40 mg/kg) or combination treatment. The mean ± SEM is shown. **B** Image of the tumours extracted from mice after 3 weeks of treatment separated into each treatment group. **C** Ki67 immunohistochemistry staining was performed on tumour tissue post treatment and the number of cells stained per field quantified. The mean ± SD is shown *p ≤ 0.05, **p ≤ 0.01 and ***p ≤ 0.001 (two-tailed *t*-test).

The in vivo anti-tumour activity of CP-673451 was evaluated using a U87 xenograft GBM mouse model (Fig. 5A). Treatments were administered as follows: DMSO control, TMZ (25 mg/kg/day), CP-673451 (40 mg/kg/day) or combination of TMZ (25 mg/kg/day) and CP-673451 (40 mg/kg/day) (Fig. 5B). Tumour volumes from mice in all three treatment groups were significantly reduced compared to the control group (620.9 ± 224.0 mm^3^): TMZ: 16.75 ± 5.4 mm^3^ (*p* ≤ 0.01), CP-673451: 35.9 ± 16.9 mm^3^ (*p* ≤ 0.01) and TMZ + CP-673451: 6.06 ± 2.3 mm^3^ (*p* ≤ 0.01). Tumour volumes from the combination treatment group were significantly reduced compared to both singular treatments.

Tumour samples were stained for proliferation marker Ki67 (Fig. 5C). The number of Ki67-positive cells per field was significantly reduced in all treatment groups compared to the control group (804 ± 282.0): TMZ: 43.3 ± 2.5 (*p* ≤ 0.01), CP-673451: 352.4 ± 115.6 (*p* ≤ 0.05) and TMZ + CP-673451: 36.2 ± 12.1 (*p* ≤ 0.001). There were significantly fewer ki67-positive cells in the TMZ + CP-673451 group compared to CP-673451 treatment alone. Altogether it appeared that CP-673451 treatment can improve the inhibitory effect of TMZ on tumour growth in vivo.

### Transcriptomic analysis reveals CP-673451 may affect KRAS and TNFα/NF-κB signalling mechanisms in U87 GBM cells

**Fig. 6:**
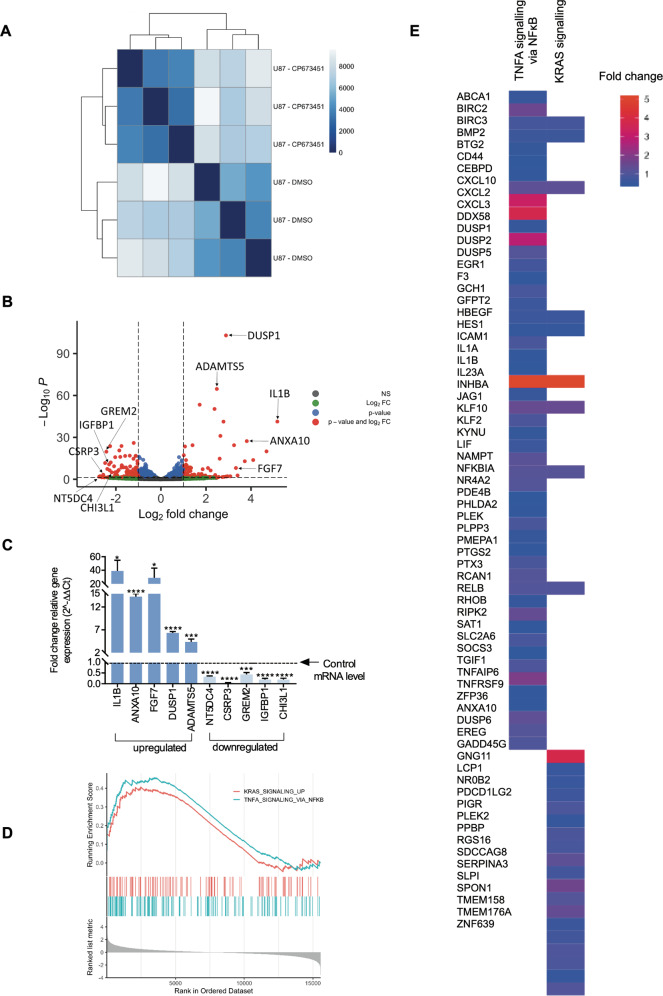
Transcriptomic analysis reveals CP-673451 may affect KRAS/NF-κB signalling mechanism in U87 GBM cells. **A** Hierarchical clustering analysis was performed using DESeq2. Dark to light colour code refers to the distance metric used for clustering. Dark blue represents the maximum correlation. **B** Volcano plot illustrating the Log2 fold change of genes that are altered upon CP-673451 treatment of U87 cells. The Log10 of *p* value, for significance in fold change, is plotted on the y-axis. Points on the plot refer to genes and are coloured according to significance. Red are genes significant by both *p* value and log2 fc, blue are significant only by *p* value but not log2 fc, green are significant only by log2 fc, grey are NS. **C** qRT-PCR performed on U87 cells treated with CP-673451 for 48 h validating genes identified from RNAseq as being upregulated (*n* = 5) and downregulated (*n* = 5). All genes show significance compared to control levels. **D** GSEA was performed to determine whether treatment of U87 cells with CP-673451 altered key Hallmark pathways curated by mSigDB. Genes predicting pathway activation of the KRAS and TNFA-NFκB signalling were significantly upregulated in U87 cells treated with CP-673451 in comparison to DMSO (FDR ≤ 0.1). **E** Heatmap from GSEA analysis illustrating expression of genes within the leading edge of the KRAS and TNFA-NF-κB Hallmark signatures. The mean ± SD of *n* = 3 independent experiments is shown *p ≤ 0.05, **p ≤ 0.01, ***p ≤ 0.001 and ****p ≤ 0.0001 (two-tailed *t*-test).

To decipher the signalling mechanism underlying the effect of CP-673451 on GBM cell differentiation, RNA-seq was performed on U87 GBM cells treated with CP-673451 for 48 h (compared to DMSO control). Whole transcriptome correlation matrix showed a high similarity between the replicate samples (Fig. 6A). DESEQ2 based differential gene expression analysis was implemented to study the transcriptional impact of inhibiting PDGF-Rα/β. Treatment with CP-673451 induced subtle alterations in the transcriptome of U87 GBM cells with the significant increased expression of 51 genes compared to DMSO control (padj < = 0.05, log2FC > = 1). CP-673451 treatment induced a significant downregulation of 54 genes compared to DMSO-treated U87 GBM cells (padj < = 0.05, log2FC < = −1). Differentially expressed genes are illustrated in a volcano plot (Fig. 6B) (< = 0.05 and log2FC > = |2 | ). Five genes that were found to be significantly upregulated (IL1B, ANXA10, FGF7, DUSP1 and ADAMTS5) and five genes that were significantly downregulated (NT5DC4, CSRP3, GREM2, IGFBP1 and CHI3L1) were chosen for validation using qRT-PCR, based on their low p-values and level of expression compared to DMSO control (Fig. 6C).

To further examine the signalling mechanisms altered by CP-673451 treatment of U87 GBM cells, Hallmark gene sets were leveraged. U87 GBM cells upon treatment with CP-673451 significantly activated Hallmark KRAS signalling (Enrichment Score = 0.40, *p*-value = 0.003) and Hallmark NF-κB signalling (Enrichment Score = 0.45, *p*-value = 0.00019) in comparison to U87 GBM cells treated with DMSO (Fig. 6D). The expression of the genes within the leading edge of the KRAS and TNFA-NF-κB signatures from GSEA are plotted (Fig. 6E). Taken together, the RNA-seq data revealed that the observed pro-differentiation effect of CP-673451 in GBM cells could be linked to the activation of KRAS- and TNFα/NF-κB-dependent signalling mechanisms.

### DUSP1 drives the pro-differentiation effect of CP-673451 treatment via inhibition of p38^MAPK^ in U87 GBM cells and G179 GSCs

Results from the RNA-seq analysis (Fig. 6) determined that the neuroprotective agent dual-specificity phosphatase 1 (DUSP1) (also known as mitogen-activated protein kinase phosphatase 1 (MKP-1)), able to induce neurite outgrowth in neurons, was significantly increased 2.9 fold (*p* = 5.33 × 10^−100^) in CP-673451 treated U87 GBM cells compared to control [32]. Further qRT-PCR validated the significant increase of DUSP1 gene expression in U87 GBM cells and G179 GSCs treated with 1 μM CP-673451, compared to levels in the DMSO control (6.4 ± 0.34 (*p* ≤ 0.0001), 2.5 ± 1.0 (ns), respectively) (Supplementary Fig. 4A, B)

DUSP1 gene silencing and inhibition using DUSP 1/6 inhibitor BCI were performed to confirm its effect on neurite-like process outgrowth in GBM cells and GSCs. Reverse transfection was performed on U87 GBM cells with two DUSP1 siRNA (SMPs 1 and 4) simultaneously at 50 nM for 24 h. G179 GSCs were treated by reverse transfection with 100 nM DUSP1 siRNA (SMPs 3 and 4) simultaneously for 48 h. DUSP1 KD was validated by qRT-PCR in U87 GBM cells and G179 GSCs 62% (0.38 ± 0.04 fc (*p* ≤ 0.0001)) and 45% (0.55 ± 0.14 fc (*p* ≤ 0.001)) compared to the control siRNA (siCTRL) (Supplementary Fig. 4). Western blotting was performed to measure the protein expression levels of DUSP1 in U87 GBM cells and G179 GSCs (Supplementary Fig. 4C, D) treated with DMSO, 1 μM CP-673451, siCTRL or siDUSP1 with DMSO or 1 μM CP-673451, and 1 μM DUSP1 inhibitor BCI with DMSO or 1 μM CP-673451. DUSP1 protein expression was shown increased in U87 GBM cells and G179 GSCs upon treatment with CP-673451. Both DUSP1 KD and BCI treatment, reduced DUSP1 expression in both non-treated and CP-673451-treated cells (Supplementary Fig. 4).

**Fig. 7:**
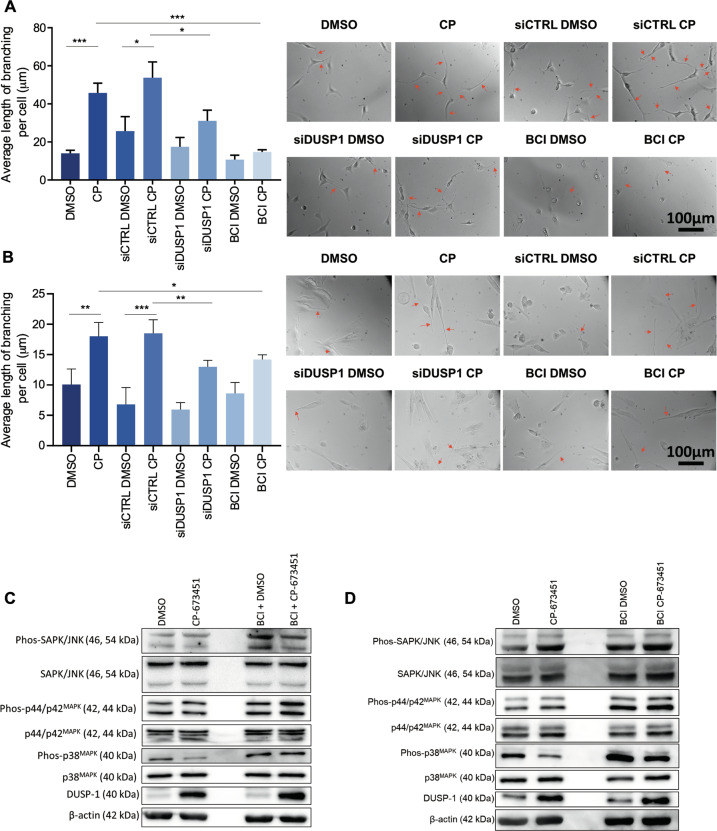
DUSP1 drives the pro-differentiation effect of CP-673451 treatment via inhibition of p38^MAPK^ in U87 GBM cells and G179 GSCs. **A**, **B** Average length of neurite-like process per cell was determined for U87 GBM cells and G179 GSCs treated with DMSO, CP-673451, siCTRL DMSO or CP-673451, siDUSP1 DMSO or CP-673451 and BCI DMSO or CP-673451 for 24 h (ordinary one-way ANOVA). Representative images shown, red arrows indicating neurite-like processes. Images taken at 20x mag. Scale bar, 100 μm. **C**, **D** Western blotting analysis of phosphorylated and total SAPK/JNK, phosphorylated and total p44/p42^MAPK^, phosphorylated and total p38^MAPK^, DUSP1 and β-actin (loading control) in U87 GBM cells and G179 GSCs treated with DMSO or CP-673451 and BCI DMSO or CP-673451 for 24 h. Representative images shown. The mean ± SD of *n* = 3 independent experiments is shown *p ≤ 0.05, **p ≤ 0.01, ***p ≤ 0.001 and ****p ≤ 0.0001 (ordinary one-way ANOVA).

The average neurite-like process length per cell was quantified in U87 GBM cells and G179 GSCs upon DUSP1 KD or BCI treatment (Fig. 7). Cells were treated with DMSO or 1 μM CP-673451, siCTRL or siDUSP1 with DMSO or 1 μM CP-673451 and 1 μM BCI with DMSO or 1 μM CP-673451. Combination of CP-673451 treatment and DUSP1 KD induced a reduction in the average neurite-like process length per cell compared to CP-673451 treatment alone in U87 GBM cells (42% (31.1 ± 5.6 μm *vs* 53.8 ± 8.27 μm (*p* ≤ 0.01)) and G179 GSCs (30% (13.0 ± 1.1 µm vs 18.5 ± 2.2 µm (*p* ≤ 0.01)) (Fig. 7A, B). In addition, CP-673451/BCI co-treatment appeared to decrease neurite-like process outgrowth in both U87 GBM cells and G179 GSCs compared to CP-673451 alone 68% (14.7 + 1.17 μm vs 45.8 ± 5.2 μm (*p* ≥ 0.001)) and 21% (14.2 ± 0.8 µm vs 18.0 ± 2.3 μm (*p* ≤ 0.05)), respectively (Fig. 7A, B).

Further validation of the effects of DUSP1 on neurite-like process outgrowth in GBM cells was performed through overexpression of the gene. Cells were transfected with 1 ng/µl control pCMV6 empty vector or DUSP1 plasmid for 48 h. Overexpression was validated through qRT-PCR in U87 GBM cells (8.3 ± 1.5-fold increase (*p* ≤ 0.05)) and G179 GSCs (3.7 ± 1.4 fold increase (*p* ≤ 0.05)) compared to control (Supplementary Fig. 5A, B). In the same way, upon overexpression, protein expression was found to increase 9.2 ± 4.6 (*p* ≤ 0.05) fold in U87 GBM cells and 30.8 ± 13.1 fold (*p* ≤ 0.05) in G179 GSCs compared to control (Supplementary Fig. 5C, D). In U87 GBM cells, transfection with the DUSP1 plasmid increased the average neurite-like process length by 61% compared to the control 21.4 ± 0.4 µm vs 13.3 ± 2.8 μm (*p* ≤ 0.01). In G179 GSCs, transfection with the DUSP1 plasmid increased the average neurite-like process length by 47% compared to the control 7.9 ± 0.3 µm vs 5.4 ± 0.6 μm (*p* ≤ 0.01) (Supplementary Fig. 5E, F).

Activation of SAPK/JNK, p44/p42^MAPK^ and p38^MAPK^, known potential mediators of DUSP1 signalling, was investigated through western blotting in U87 GBM cells and G179 GSCs treated with CP-673451 and BCI compared to CP-673451 treatment alone [32]. Levels of phosphorylated p38^MAPK^ were observed reduced in both U87 GBM cells and G179 GSCs upon treatment with CP-673451 (0.85 ± 0.09 (*p* ≤ 0.05) and 0.72 ± 0.16 (*p* ≤ 0.05), respectively). Co-treatment with DUSP1 inhibitor BCI abrogated this decrease in p38^MAPK^ phosphorylation (Fig. 7C, D).

**Fig. 8:**
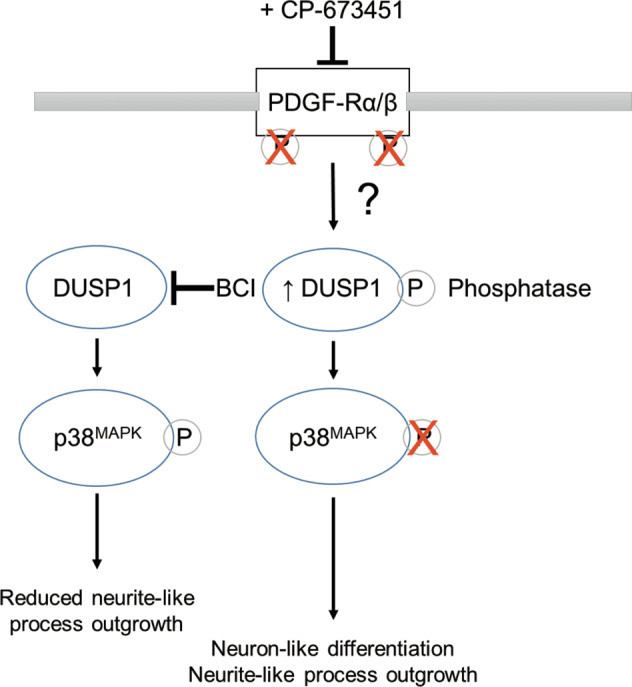
PDGF-Rα/β inhibition by CP-673451 induces differentiation of glioblastoma cells via DUSP1/p38^MAPK^ pathway activation. Diagram of potential mechanism of action of CP-673451 in GBM cells.

Taken together, our data suggest that treatment of GBM cells with CP-673451 inhibits PDGF-Rα/β, inducing the upregulation of phosphatase DUSP1, which reduces levels of phosphorylated p38^MAPK^, ultimately leading to the onset of neuron-like differentiation and consecutive neurite-like process formation (Fig. 8) [32].

## Discussion

GBM is in urgent need of novel therapies to significantly improve patients’ survival and quality of life [2, 3, 5]. Indeed, despite several advancements in treatment options, most of them, including anti-VEGF therapy with bevacizumab and tumour treating fields, are still debated [33, 34]. The emergence of differentiation therapeutic strategies has brought new hope through proposing to induce tumour cell differentiation into normal cells, thus making cancer growth more manageable. Here, we report how the PDGF-R inhibitor CP-673451 could be used to this purpose to treat GBM by initiating neuronal differentiation in tumour cells, consecutively reducing tumour development.

Our data revealed that treatment of GBM cells and GSCs with CP-673451 can induce outgrowth of neurite-like processes and expression of the neuronal marker NEFL, while inhibiting proliferation and invasion in vitro. Neurite outgrowth has been historically recognised as a marker of neuronal differentiation and slowing cell proliferation is a well described trait of differentiating cells, including neurons. Along with neurofilament proteins, including NEFL, β3-tubulin (TUBB3) is essential to the growing neurites of the developing neurons [17, 35, 36]. Intriguingly, in the present study, the impact of CP-673451 treatment on the expression of TUBB3 was limited, as compared to what was observed on NEFL expression. While unexpected, such observation might suggest that GBM cells might not be differentiating into full grown neurons upon treatment with CP-673451. Accordingly, such capacity of GBM cells to adopt more differentiated cell properties while partially maintaining a tumour phenotype has in recent years been reported in studies showing GBM cells able to differentiate into endothelial-like cells to maintain neo-angiogenesis in the tumour microenvironment for their own invasion [7, 37]. Our results hence suggest that CP-673451 treatment of GBM cells differentiate them into an intermediate post-mitotic/partially differentiated ‘neuron-like’ state.

Further supporting this observation, our RNA-seq analysis identified DUSP1 expression to be significantly upregulated in GBM cells upon treatment with CP-673451. DUSP1 has been reported to be essential in the early hours of neuronal differentiation during embryogenesis. Its activity is required for both limiting cell proliferation and ensuring proper neurite outgrowth. DUSP1 is also directly implicated in the maintenance of neuronal integrity [38]. Previous studies have determined that recovery of DUSP1 expression is neuroprotective and neural mediators such as nerve growth factor (NGF) [39] and brain-derived neurotrophic factor (BDNF) [40] induce neuronal survival and differentiation through increased DUSP1 expression. More specifically, DUSP1 expression has been shown to correlate with synaptic activity and is required for proper dendritic growth and axonal arborisation [40]. Accordingly, Collins et al. identified that overexpressing DUSP1 caused greater morphological complexity in dopaminergic rat neurons, characterised by longer neurites [32]. Authors also showed that treatment of these neurons with neurotoxin 6-hydroxydopamine (6-OHDA) in vitro inhibited neurite outgrowth through activation of p38^MAPK^, one of DUSP1 down-stream targets, also including p44/p42^MAPK^ (ERK1/2) and SAPK/JNK [32, 38, 41]. In the current study, we report that treatment with CP-673451 may trigger neuron-like differentiation in GBM cells and GSCs *via* p38^MAPK^ inhibition due to DUSP1 activity. Interestingly, a separate study reported that high DUSP1 levels correlate with increased GBM patient survival. Authors revealed that overexpression of DUSP1 in GSCs impedes self-renewal and induces differentiation *via* deactivation of p38^MAPK^ in vitro, reducing tumourigenicity and increasing sensitivity to TMZ therapy [42]. Here, our own in vivo data accordingly showed that treatment with CP-673451 significantly improves response to TMZ in treated animals.

Taken together, these results thus suggest that CP-673451 treatment could hold great promise as part of a novel therapeutic strategy against GBM. CP-673451 is a low molecular weight kinase inhibitor (molecular weight = 417.52) [26] with the potential capacity to diffuse freely through the BBB, although further in vitro and in vivo studies are certainly needed in order to measure the BBB permeability of this compound and whether it reaches the tumour-site at a physiologically relevant dose [43, 44]. As mentioned above, inducing tumour cell differentiation, even partially, could make cancer growth more manageable and consecutively an ‘easier’ target for more conventional strategies, including surgery and chemotherapies. This appears to be a very crucial point for GBM specifically, known to rapidly diffuse into the brain tissue as the tumour develops [45, 46]. Theoretically, a potential therapeutic strategy sequence including differentiation therapy with CP-673451 could thus start with the original debulking surgery, followed by CP-673451 treatment, and TMZ chemotherapy: this would allow CP-673451 to trigger differentiation of any potential residual GBM or GSCs left by the surgery, reducing risk of treatment resistance and recurrence leading to patient relapse. In addition, one could speculate that such strategy could allow for reducing the use of more conventional and ‘aggressive’ chemotherapies, which would increase patient quality of life [47, 48]. Furthermore, CP-673451 treatment could also be of great benefit for tackling neurological disorders such as Alzheimer’s, Huntington’s and Parkinson’s diseases, in which DUSP1 dysregulation has been reported [38]. Nevertheless, these observations are only speculative for now and would require further future investigations.

To our knowledge, this is the first time such an effect of PDGF-R inhibition on GBM cell differentiation *via* DUSP1 is being reported. Yet, our present investigation presents some limitations that would need to be addressed in follow-up studies. Among these, the upstream mechanism between PDGF-R inhibition and upregulation of DUSP1 is yet to be elucidated. However, our RNA-seq data demonstrated a significant enrichment of genes linked to the NF-κB pathway upon treatment of GBM cells with CP-673451. Accordingly, the promoter region of the DUSP1 gene contains binding sites for NF-κB [49]. In addition, further work should look into how CP-673451 could differently affect distinct GBM subtypes, which could provide insights on determining the GBM patients that could benefit the most from a more personalised therapeutic strategy [2, 45]. Lastly, further experimentation into potential resistance mechanisms of CP-673451 could be a very interesting avenue to explore in future studies, as it has already been reported that GBM cells can recruit different compensatory pathways, including ERBB3, IGF1R, TGFBR2 and IGF1R-mediated signalling, in order to induce resistance to PDGF-R inhibition [50, 51].

Altogether, this study has identified a KI, CP-673451, able to induce differentiation in GBM cells, with the potential to target GSCs, which are known to be directly implicated in GBM therapeutic resistance and inevitable recurrence. CP-673451 treatment could thus refine therapeutic strategies against GBM, through reducing side effects and enhancing response to current therapies, consequently improving patients’ quality of life.

## Materials and methods

### Cell culture

Cells were maintained at 37 °C in a humidified 5% CO_2_ atmosphere. Astrocytes and established human GBM cell lines were purchased from the American Type Culture Collection (ATCC). Astrocytes (AS) were seeded onto flasks and plates pre-treated with 2 μg/cm^2^ poly-L-lysine (Sigma-Aldrich) and required Astrocyte growth medium (Sigma-Aldrich) 10% foetal bovine serum (FBS) (Sigma-Aldrich) and 100 units ml^−1^ penicillin, 100 μg ml^−1^ streptomycin and L-glutamine (1% PSG) (Sigma-Aldrich). U87 and U138 cells required minimum essential medium (MEM) 10% FBS and 1% PSG. LN229 cells required Dulbecco’s modified eagle medium (DMEM) 10% FBS and 1% PSG.

Patient-derived GSCs (GS090, G166 and G179) were a kind gift from Dr. Angela Bentivegna, University of Milan-Bicocca, Italy and Dr. David Nathanson, University of California, Los Angeles, US. GSCs were isolated from GBM tumour samples following local ethical board approval. GSCs were maintained as neurospheres in DMEM/F12 Ham (Sigma-Aldrich), 1% B27 without vitamin A (Gibco), 1% Glutamax (Gibco) and 1% PSG with a mix of growth factors (Final concentrations; Heparin 5 μg/ml, HuEGF 5 ng/ml and HuFGF-β 20 ng/ml). Medium was changed twice a week. GBM cell lines and astrocytes were detached at confluence using trypsin/EDTA (Sigma-Aldrich). GSCs were disassociated using TrypleE express enzyme (Gibco) and separated into single cells through a 70 μm cell strainer (Starstedt). All cells were tested for mycoplasma at the beginning of the study.

### Kinase inhibitor treatment/screening

Cells were counted using the automated cell counter Countess (Invitrogen), seeded (2 × 10^3^ cells per well) into a 96-well plate and treated with 1 μM of 47 KIs from a library of 378 (SelleckChem) for 24 h in 10% FBS. The automated liquid handler Biomek 4000 was used for performing this screening (Beckman Coulter). Dimethyl sulfoxide (DMSO) was used as a vehicle control. GSCs were treated with 10% FBS to enhance cell attachment. Following 24 h (GBM cell lines) and 48 h (GSCs) treatment with KIs, images of the cells were taken, and length of neurite-like process was measured as described below.

### Light microscopy and neurite-like process length analysis

Light microscopic images were taken at 20x magnification (mag) using the Olympus IX71 microscope with Micromanager software. The length of long thin extensions termed ‘neurite-like processes’ were analysed. Three technical repeats were performed with nine images taken per well and all neurite-like processes analysed. The semi-automatic Fiji (ImageJ) plugin ‘simple neurite tracer’ was used to measure the lengths of the neurite-like processes. The total lengths of neurite-like processes per well were quantified and normalised by the total number of cells per well to calculate the average length of neurite-like process per cell. Graphs display the mean ± SD of at least three independent experiments, representative images shown, red arrows indicating neurite-like processes.

### Cell proliferation assay

Cells were seeded (2 × 10^3^ or 5 × 10^3^ cells per well) into a 96-well plate with 10% FBS 1% PSG for 24 h before the treatments were applied. Cells were then treated with 0, 1, 5 or 10 µM of the stated KIs. Following 24 h (GBM cell lines) or 48 h (GSCs) treatment, cells were fixed with 4% paraformaldehyde (PFA) in 1x phosphate buffered saline (PBS) (Sigma-Aldrich) and incubated at room temperature (temp) for 30 min. PFA was removed and plates left to dry at room temp for 10 min. They were then washed once with PBS and stained with 0.1% crystal violet solution at room temp for 30 min. The solution was then aspirated and washed in 1x PBS twice and water once and left open overnight to dry. When ready to read, 50 μl of 10% acetic acid was added to each well, plates were agitated on a plate rocker for 20 min and absorbance was recorded at 600 nm using a GloMax Explorer reader (Promega). Graphs show the mean ± SD of at least three independent experiments.

### Cell viability assay

Cells were seeded (2 × 10^3^ cells per well) into a 96-well plate with 10% FBS 1% PSG for 24 h before the treatments were applied. Cells were then treated with DMSO or 1 µM of the stated KIs. Following 24 h (GBM cell lines) or 48 h (GSCs) treatment, 10 μl of water-soluble tetrazolium salts-1 (WST-1) reagent was added to each well and incubated at 37 °C, 5% CO_2_ for 2 h. The plate was then agitated on a plate rocker at room temp for 1 min and the absorbance recorded at 450 nm using a GloMax Explorer reader (Promega). Graphs show the mean ± SD of at least three independent experiments.

### RNA extraction, library construction and RNA-sequencing

U87 GBM cells were treated with DMSO or CP-673451 (CP) for 48 h and RNA was purified following PureLink RNA Mini Kit (Life technologies) protocol, as described before [52]. All conditions were analysed using three biological replicates. RNA samples were then quantified using a Qubit 2.0 (Life Technologies, CA, USA) fluorimeter from Invitrogen with a high sense RNA kit. The quality of RNA was assessed and confirmed using the RNA Pico 6000 kit. The high-quality RNA was then used for library preparation using the RNA hyper prep kit with riboerase (KAPA Biosystems Cat. No. KK8560) according to the manufacturer’s recommendation and were fragmented at 85 °C for 4.5 min. The libraries were amplified using eight cycles of PCR and were ligated with the NEXTflex-96 DNA barcodes (Bio Scientific). The library was quantified using the Qubit DNA high sense kit, and its quality was confirmed using Bioanalyzer (DNA high sense kit). Libraries were then pooled and diluted to a final concentration of 2 nM. The pooled and diluted library were denatured along with 1% PhiX spike as per recommendation from Illumina. Library was loaded onto the NextSeq 550 v2 mid-output 150 cycle kit.

### Raw data processing, alignment analysis, and identification of differentially expressed genes

High-quality clean reads were obtained by trimming the adaptor sequences and removing reads that contained poly-N or were of low-quality from the raw data using the fastX tool kit (v 0.0.14) (http://hannonlab.cshl.edu/fastx_toolkit/license.html). The quality of reads were confirmed using the fastqc tool kit (v 0.11.5) (http://www.bioinformatics.babraham.ac.uk/projects/fastqc/), and only high-quality clean read were used for down-stream analyses. The high-quality reads were mapped to the ENSEMBL built GRCH37 using the STAR aligner (v2.5.3a) [53] with the ENCODE options as described in the STAR manual. Mapped reads were then used to estimate the read counts for each gene using the Summarise Overlaps function provided with the R package DESeq2 [54]. Differential gene expression analysis was performed using DESeq2 non-interaction model. The Benjamini-Hochberg corrected *p* value (*P*_adj_ ≤ 0.05) and Log2 fold change ≥ |1| were used as the threshold to screen significance of differentially expressed genes (DEGs). Gene set enrichment function from the enrichplot package (https://github.com/YuLab-SMU/enrichplot) was used to perform gene set enrichment analysis for Hallmark gene sets and CAHOY CNS neural cell type gene sets deposited in the MSIGDB (https://www.gsea-msigdb.org/gsea/msigdb/index.jsp).

### Gene expression analysis by qRT-PCR

Cells were seeded (5 × 10^5^ cells per T75 cm^2^ or 1 × 10^5^ per well into a 6-well plate) and were treated with drug/control or target/control siRNA plasmid/control for the concentration and time stated. Cells were lysed, RNA was then purified following PureLink RNA Mini kit (Life technologies) protocol. Reverse transcription was carried out using a cDNA synthesis kit (Applied Biosystems). Taqman (Applied Biosystems) or SYBR Green (Applied Biosystems) gene expression master mix and synthesised cDNA was mixed with primers (Applied Biosystems) (Tables 2 and 3) and run on a StepOnePlus Real-Time PCR system (Applied Biosystems). Data were evaluated using StepOnePlus software (Applied Biosystems). A minimum of three technical repeats were performed for each experiment and results normalised with internal control glyceraldehyde-3-phosphate dehydrogenase (GAPDH). Graphs show the mean ± SD of at least three independent experiments.

**Table 2:** Primers separated into cell type used in qRT-PCR.

Cell type	Primer	Assay ID (Applied Biosystems)
Astrocytic	GFAP	Hs00909233_m1
	ALDH1L1	Hs01003842_m1
Neuronal	TUBB3	Hs00801390_s1
	NEFL	Hs00196245_m1
Oligodendrocytic	OLIG2	Hs00300164_s1
	CSPG4	Hs00361541_g1
Stem cell	Nestin	Hs04187831_g1
	PROM1 (CD133)	Hs01009259_m1
Mesenchymal	CD44	Hs01075864_m1
	VEGFA	Hs00900055_m1
Control	GAPDH	Hs99999905_m1

**Table 3:** Primers used for the delineation of signalling pathways in qRT-PCR.

Primer	Assay ID/target Sequence	Source
Hs_PDGFRA_1_SG	NM_006206	Qiagen
Hs_PDGFRB_1_SG	NM_002609	Qiagen
DUSP1	NM_004417	Qiagen
GAPDH forward	5′-CAGCAAGAGCACAAGAGGAAG-3′	Eurofins
GAPDH reverse	5′-TGGTACATGACAAGGTGCGG-3′	Eurofins

### Western Blotting

5 × 10^5^ cells were seeded per T75cm^2^ flask and incubated for 24 h before treatment with: 1 µM CP-673451 for 0, 15 min, 1, 4, 24 and 48 h *vs* DMSO (control) in 0% FCS medium; 1 μM CP-673451 and 1 μM DUSP1 inhibitor BCI ((E)−2-benzylidene-3-(cyclohexylamino)−2,3-dihydro-1H-inden-1-one) (Merck Millipore) *vs* 1 μM BCI and DMSO *vs* 1 μM CP-673451 *vs* DMSO (control) for 24 h in 10% FCS medium. Cells were collected and protein extracted using RIPA buffer (Sigma) including fresh protease and phosphatase inhibitors (Roche). Standard western blotting protocol was performed [55] with a 12% tris-glycine gel, semi-dry transfer onto a nitrocellulose membrane (Amersham), blocked for 1 h at room temp with 5% (w/v) non-fat milk or 5% (w/v) bovine serum albumin (BSA) (Sigma-Aldrich) in tris-buffered saline (TBS) containing 0.1% (v/v) Tween-20 (TBST). Primary antibodies (anti-phosphorylated (phos)-PDGF-Rα (Tyr849)/ PDGF-Rβ (Tyr857) (Cell Signaling 1:1000 dilution), anti-PDGF-Rα (Cell Signaling 1:1000), anti-PDGF-Rβ (Cell Signaling 1:1000), anti-DUSP1 (Cell Signaling 1:1000), anti-phos-p38^MAPK^ (Cell Signaling 1:1000), anti-p38^MAPK^ (Cell Signaling 1:1000), anti-phos-SAPK/JNK (Cell Signaling 1:1000), anti-SAPK/JNK (Cell Signaling 1:1000), anti-phos-p44/p42^MAPK^ (Cell Signaling 1:1000), anti-p44/p42^MAPK^ (Cell Signaling 1:1000) and anti-β-actin (Genscript #A00702 1:1000 dilution)) were prepared in the same solution used for blocking and incubated on a rocker at 4 °C overnight (Table 3). The following day membranes were washed three times for 5 min in TBST at room temp and incubated in secondary antibody (Polyclonal Goat Anti-Rabbit/Mouse Immunoglobulins/HRP (Dako P0447/8, 1:3000 dilution)) for 1 h at room temp followed by three more 5 min TBST washes at room temp (Table 3, 4). Chemiluminescence was observed using a UVP Chemstudio instrument (Analytik Jena) and the Vision Works software. Experiments have been repeated at least three times with representative images shown.

### Cell Invasion Assay in HA hydrogels

Cells were seeded (1 × 10^5^ cells per well) into a 96-well HA hydrogel assay plate. As per instructions, 40 µl of cell suspension was added as a drop on top of the gel, incubated for 30 min then 100 µl media was added, as previously described [45, 56]. Cells were incubated for 48 h before treatment with 0, 1, 5 and 10 µM CP-673451 with DMSO as vehicle control. Media was replaced every 2 days. Following 7 days of treatment, nine light microscopic images per well were taken at 10x mag using the Olympus IX71 microscope with Micromanager software with three technical repeats performed per condition. The areas of colonies were measured, and colonies counted (Fiji – ImageJ). Graphs display the mean ± SD of at least three independent experiments with representative images shown.

### CellTiter-Glo luminescent viability assay

Following the cell invasion assay, cell viability was assessed using CellTiter-Glo luminescent cell viability assay (Promega). Cells were incubated at room temp for 30 min before 100 µl CellTiter-Glo reagent was added to each well, agitated on a plate rocker for 2 min and incubated at room temp for 10 min. Luminescence was recorded with an integration time of 0.3 s using a GloMax Explorer reader (Promega). Graphs show the mean ± SD of at least three independent experiments.

### Gene silencing; small interfering RNA

Cells were seeded (2 × 10^3^ (U87 GBM cells) or 5 × 10^3^ (G179 GSCs) cells per well) into a 96-well plate and incubated at 37 °C, 5% CO_2_ for 24 h. Transfection was performed according to the Lipofectamine 3000 (Invitrogen) user guide. Lipofectamine and siRNA complexes were diluted separately in Opti-MEM (Gibco). These solutions were mixed and incubated at room temp for 15 min. Media was then aspirated, fresh media (10% FCS, 0% PSG) was added to the cells and the mixed siRNA/Lipofectamine/Opti-MEM solution was added dropwise. Cells were transfected for 48 h with 50 nM and 100 nM siRNA (control (CTRL), PDGF-Rα, PDGF-Rβ or a combination of PDGF-Rα and *β*) (Table 4) with three technical repeats per condition. At 48 h nine light microscopic images were taken per well at 20x mag and average length of neurite-like process per cell determined as described previously. Graphs display the mean ± SD of at least three independent experiments. For qRT-PCR experiments cells were seeded (1 × 10^5^ cells per well) into a 6-well plate, transfected as described with 50 nM and 100 nM CTRL siRNA or a combination of PDGF-Rα and PDGF-Rβ siRNA. At 48 h cells were lysed, RNA purified, cDNA synthesised and mixed with SYBR Green master mix (Applied Biosystems) plus primers (Table 3). qRT-PCR was then performed as described previously. DUSP1 knockdown (KD) experiments were performed using the same method but using reverse transfection using 50 nM CTRL siRNA or DUSP1 siRNA (SMPs 1 and 4) for 24 h in U87 GBM cells and 100 nM CTRL siRNA or DUSP1 siRNA (SMPs 3 and 4) with HiPerFect transfection reagent (Qiagen) for 48 h in G179 GSCs (Table 4) before 1 μM CP-673451 or DMSO was added for a further 24 h.

**Table 4:** siRNA for the delineation of signalling pathways used in siRNA knockdown.

siRNA	Assay ID/ Target Sequence	Source
Hs_PDGFRA_11 FlexiTube siRNA	5′-CTGGAGGGTCATTGAATCAAT-3′	Qiagen
Hs_PDGFRB_11 FlexiTube siRNA	5′-CACGATGAAAGTGGCCGTCAA-3′	Qiagen
DUSP1_1	5′-TAGCGTCAAGACATTTGCTGA-3′	Qiagen
DUSP1_3	5′-CACGAACAGTGCGCTGAGCTA-3′	Qiagen
DUSP1_4	5-CAGTTGTATGTTTGCTGATTA-3′	Qiagen
Control siRNA	Annealed, double stranded	Qiagen

### DUSP1 overexpression

Plasmid transfection was performed with 1 ng/µl pCMV6 empty vector or DUSP1 plasmid (Origene RC205220) for 48 h using transfection reagent HiPerFect. Graphs display the mean ± SD of at least three independent experiments, representative images shown.

### Xenograft model in vivo

Following local authority ethical approval by Sun Yat-sen University Animal Care and Use Committee, xenograft mouse models were developed by injecting 2 × 10^6^ U87 GBM cells subcutaneously into 6-week-old immunocompromised athymic nude mice (Vital River Laboratories). Once tumours were palpable (tumour diameter ~ 3 mm^3^), mice were randomly divided into the separate groups: DMSO control (*n* = 6), TMZ (25 mg/kg/day) (*n* = 9), CP-673451 (40 mg/kg/day) (*n* = 9) or combination of TMZ (25 mg/kg/day) and CP-673451 (40 mg/kg/day) (*n* = 9). Treatments were administered by oral gavage 5 days per week for 3 weeks after which the mice were sacrificed, tumours extracted and tumour volumes measured using the formula: (volume (mm^3^) = (length x height^2^)/2). Paraffin-embedded tumours were sectioned at a thickness of 4 mm with five tissue samples obtained per tumour. Samples were placed in a pressure cooker for 15–20 min in 0.01 M citrate buffer (pH 6.0) to remove aldehyde links formed during initial fixation of tissues. Specimens were incubated with antibodies specific for Ki-67 (1:100) overnight at 4 °C and immunodetection performed the following day using 3,3′-diaminobenzidine (DAB) (Dako) according to manufacturer’s instructions. Images were obtained using Olympus BX63 microscope (200x mag) and the number of cells per field quantified.

### Statistical analysis

Sample size was set to a minimum of three independent experiments (biological repeats) and experimental findings were reliably reproducible. GraphPad Prism was used for data analysis and graphs. All data are presented as mean ± SD. *T*-tests were used to compare control group with each treatment group. Differences were considered statistically significant at *p* ≤ 0.05. (*p ≤ 0.05, **p ≤ 0.01, ***p ≤ 0.001, ****p ≤ 0.0001).

## Supplementary information

Supplementary Figures 1–5

## Data Availability

All relevant data are available from the authors upon request.
