# Supplementary material for: PDGF-R inhibition induces glioblastoma cell differentiation via DUSP1/p38MAPK signalling
Source: Oncogene. 2022 Apr 7;41(19):2749–63. doi: 10.1038/s41388-022-02294-x (PMC9076540; doi:10.1038/s41388-022-02294-x)
Supplement: Supplementary file 1 — Supplementary Figures 1–5 [file 41388_2022_2294_MOESM1_ESM.pdf]

# Supplementary Figures and Figure legends

## Supplementary Figure 1

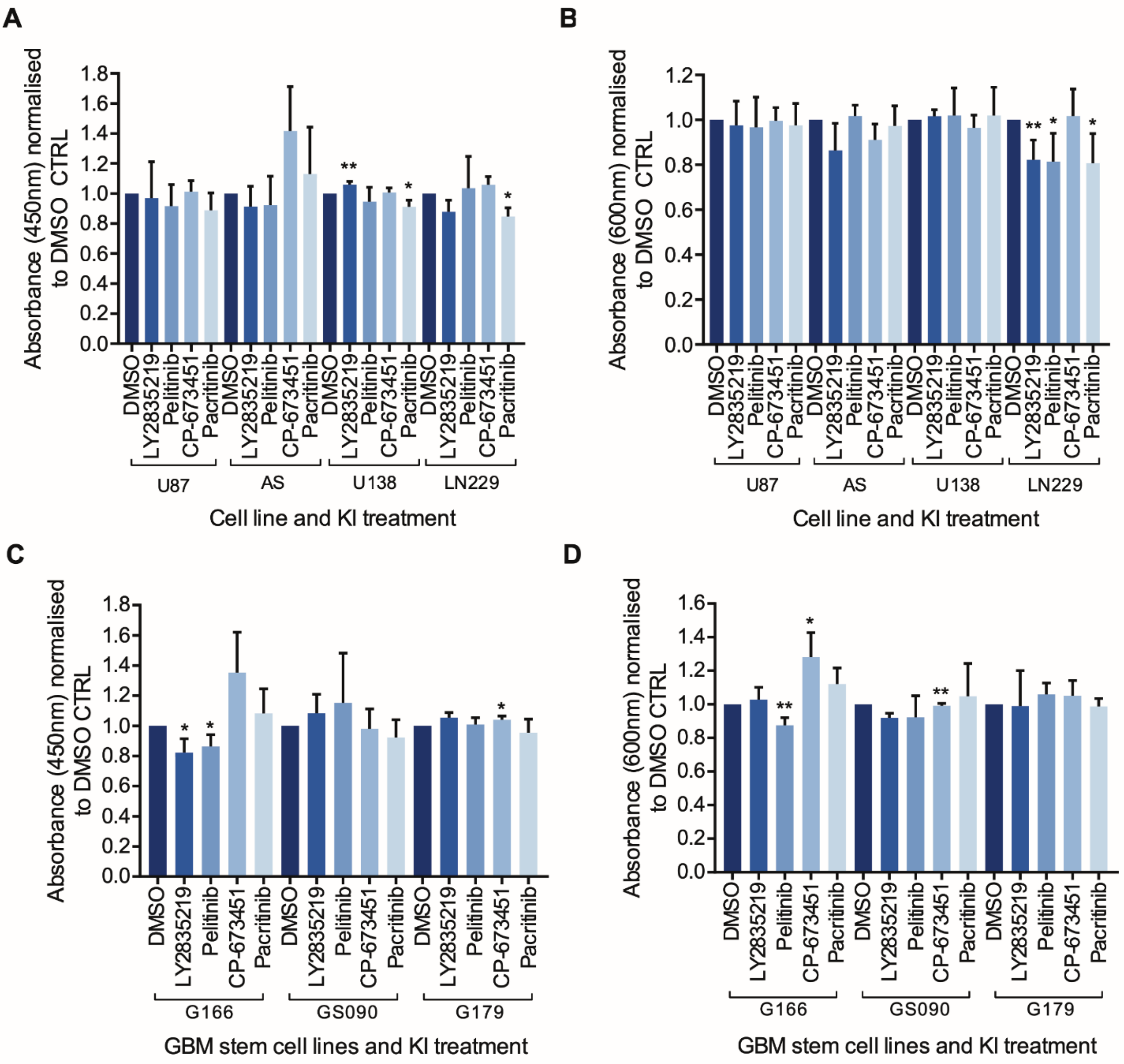


**Supplementary Figure 1: Impact of the selected kinase inhibitors on GBM cell and patient-derived GSC viability and proliferation. A** Viability assay (WST-1) was performed on normal AS and GBM cell lines U87, U138 and LN229 treated with 1µM kinase inhibitor for 24h. Values normalised to DMSO control. **B** Proliferation assay (crystal violet) was performed on normal AS and GBM cell lines U87, U138 and LN229 treated with 1µM kinase inhibitor for 24h. Values normalised to DMSO control. **C** Viability assay (WST-1) was performed on patient-derived GSCs G166, GS090 and G179 treated with 1µM kinase inhibitor for 48h. Values normalised to DMSO control. **D** Proliferation assay (crystal violet) was performed on patient-derived GSCs G166, GS090 and G179 treated with 1µM kinase inhibitor for 48h. The mean ± SD of a minimum of n=3 independent experiments is shown *p≤0.05 **p≤0.01 ***p≤0.001 (two-tailed t-test).

## Supplementary Figure 2

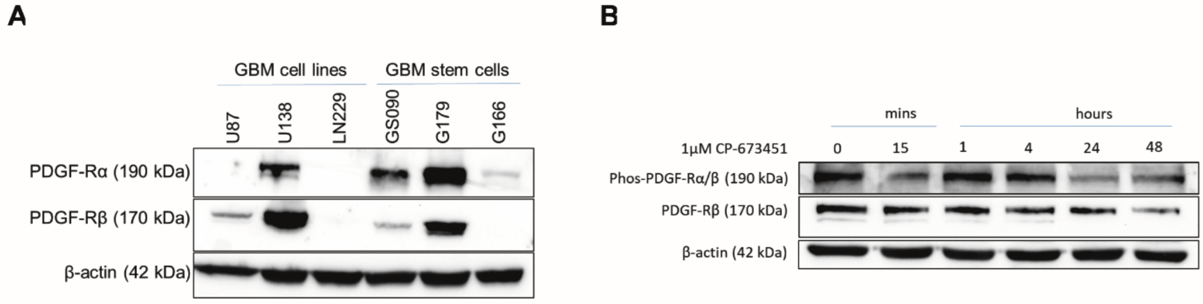


**Supplementary Figure 2: CP-673451 treatment effect on PDGF-R activation in GBM cells. A** Western blotting analysis of the expression of PDGF-Rα and PDGF-Rβ in GBM cell lines (U87, U138, LN229) and GSCs (GS090, G179 and G166). **B** Western blotting of U87 treated with 1μM CP-673451 for 0, 15mins, 1, 4, 24 and 48h and blotted for phos-PDGF-Rα (Tyr849)/ PDGF-Rβ (Tyr857) and total PDGF-Rβ normalised to loading control (β-actin).

**Supplementary Figure 3**

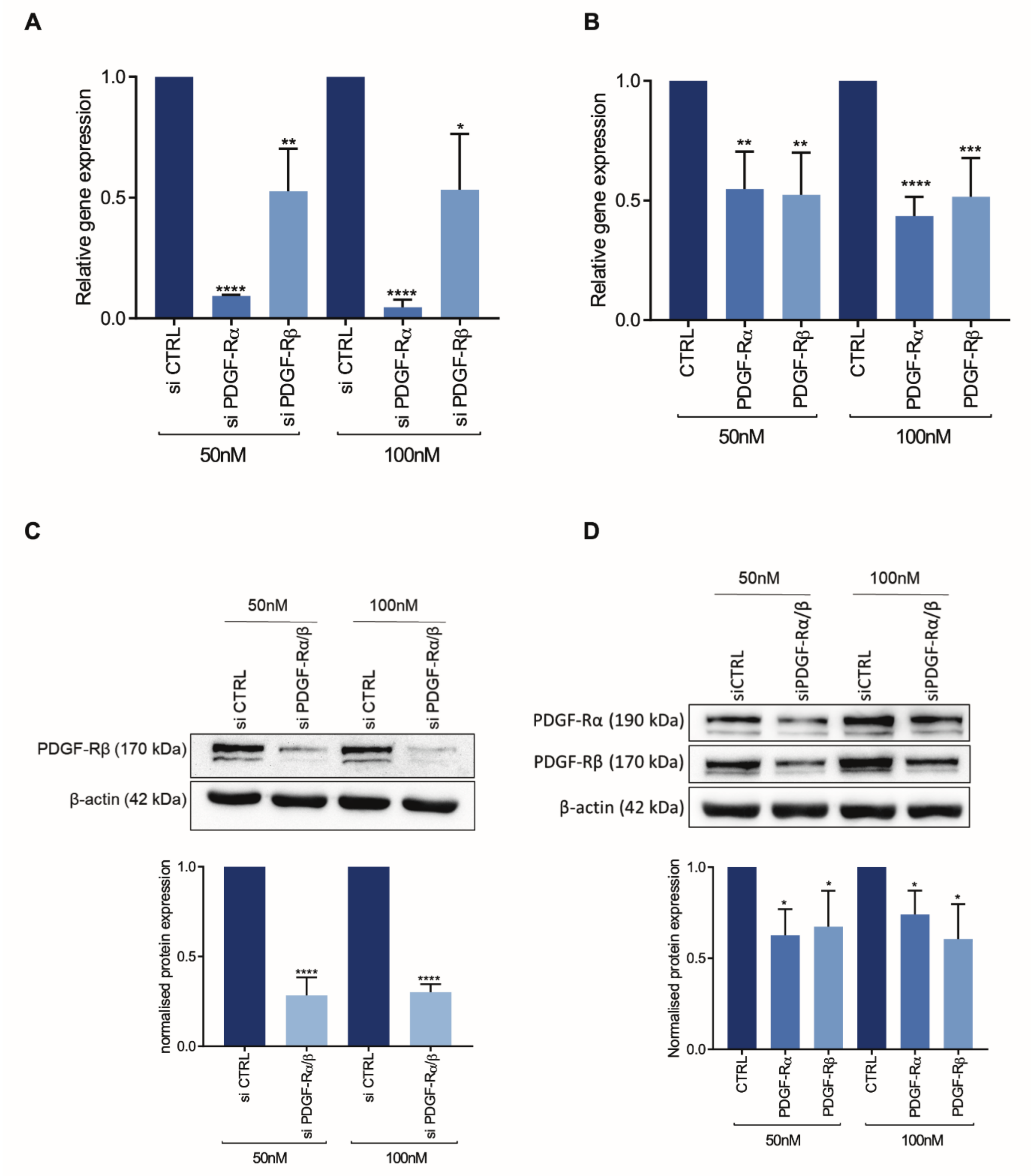


**Supplementary Figure 3: PDGF-Rα/β gene silencing in U87 GBM cells and G179 GSCs. A-B** qRT-PCR was performed measuring relative mRNA expression of PDGF-Rα and β normalised to GAPDH after U87 cells (A) and G179 GSCs (B) were treated with 50nM and 100nM siRNA CTRL or PDGF-Rα and β for 48h (fold change compared to control levels 1). **C** Western blotting analysis of PDGF-Rβ in U87 treated with 50 and 100nM siRNA CTRL or PDGF-Rα and β for 48h normalised to loading control (β-actin) and CTRL. The mean ± SD of minimum n=3 independent experiments is shown *p≤0.05 ** p≤0.01 *** p≤0.001 (two-tailed t-test). **D** Western blotting analysis of PDGF-Rα and PDGF-Rβ in G179 GSCs treated with 50 and 100nM siRNA CTRL or PDGF-Rα and β for 48h normalised to loading control (β-actin) and CTRL. The mean ± SD of minimum n=3 independent experiments is shown *p≤0.05 **p≤0.01 ***p≤0.001 (two-tailed t-test).

**Supplementary Figure 4**

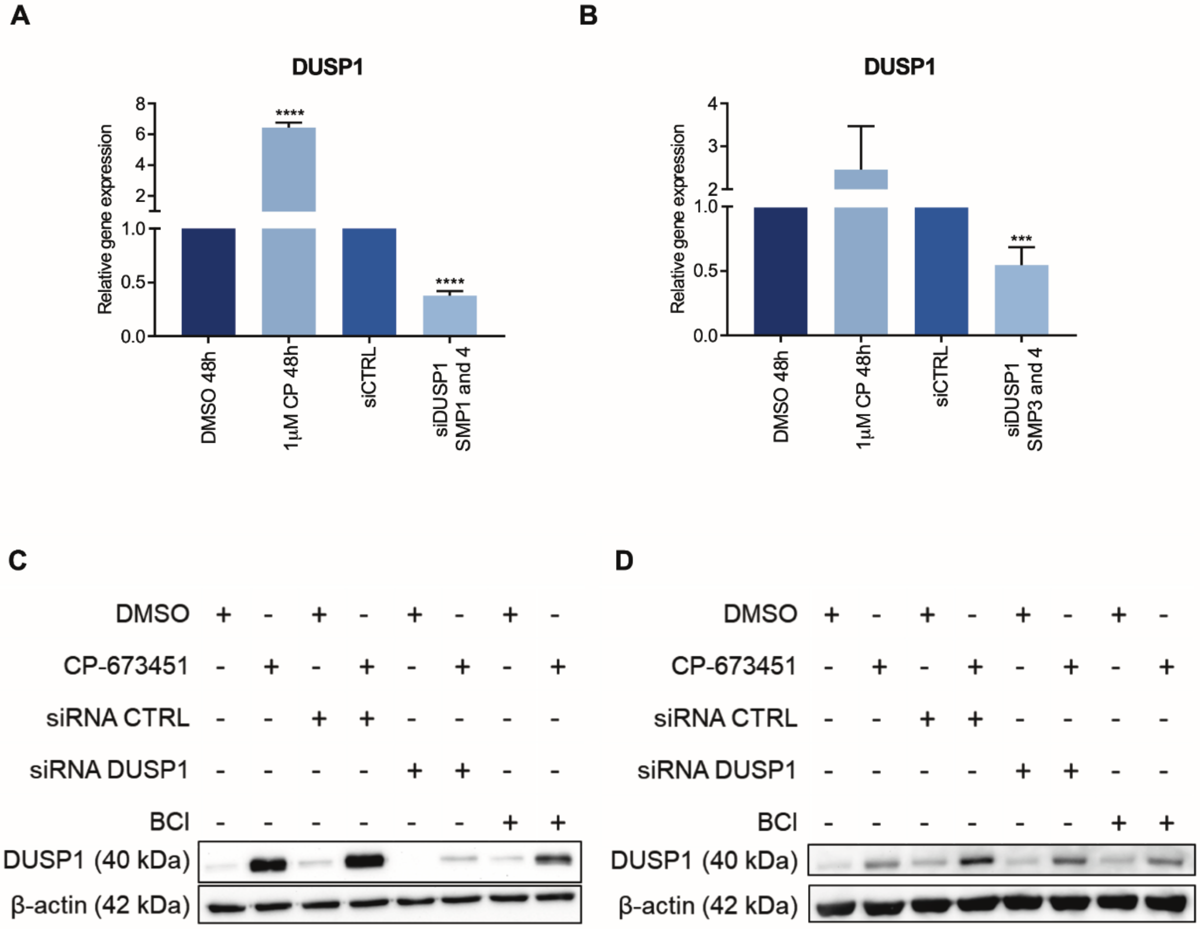


**Supplementary Figure 4: CP-673451 treatment increases DUSP1 expression in U87 GBM cells and G179 GSCs. A-B** qRT-PCR measuring relative mRNA expression levels of DUSP1 in U87 GBM cells (A) and G179 GSCs (B) treated with CP-673451 (CP) for 48h and knockdown of DUSP1 with siRNA (SMP 1 and SMP 4 for 24h and SMP3 and SMP4 for 48h, respectively) normalised to GAPDH compared to control levels. The mean ± SD of minimum n=3 independent experiments is shown *p≤0.05 **p≤0.01 ***p≤0.001 (two-tailed t-test). **C-D** Western blot illustrating protein expression of DUSP1 in U87 GBM cells (C) G179 GSCs (D) after 24h treatment with DMSO, CP-673451, siCTRL DMSO or CP-673451, siDUSP1 DMSO or CP-673451 and DUSP1 inhibitor, BCI DMSO or CP-673451 with β-actin (loading control). Representative images shown from a minimum n=2 independent experiments.

**Supplementary Figure 5**

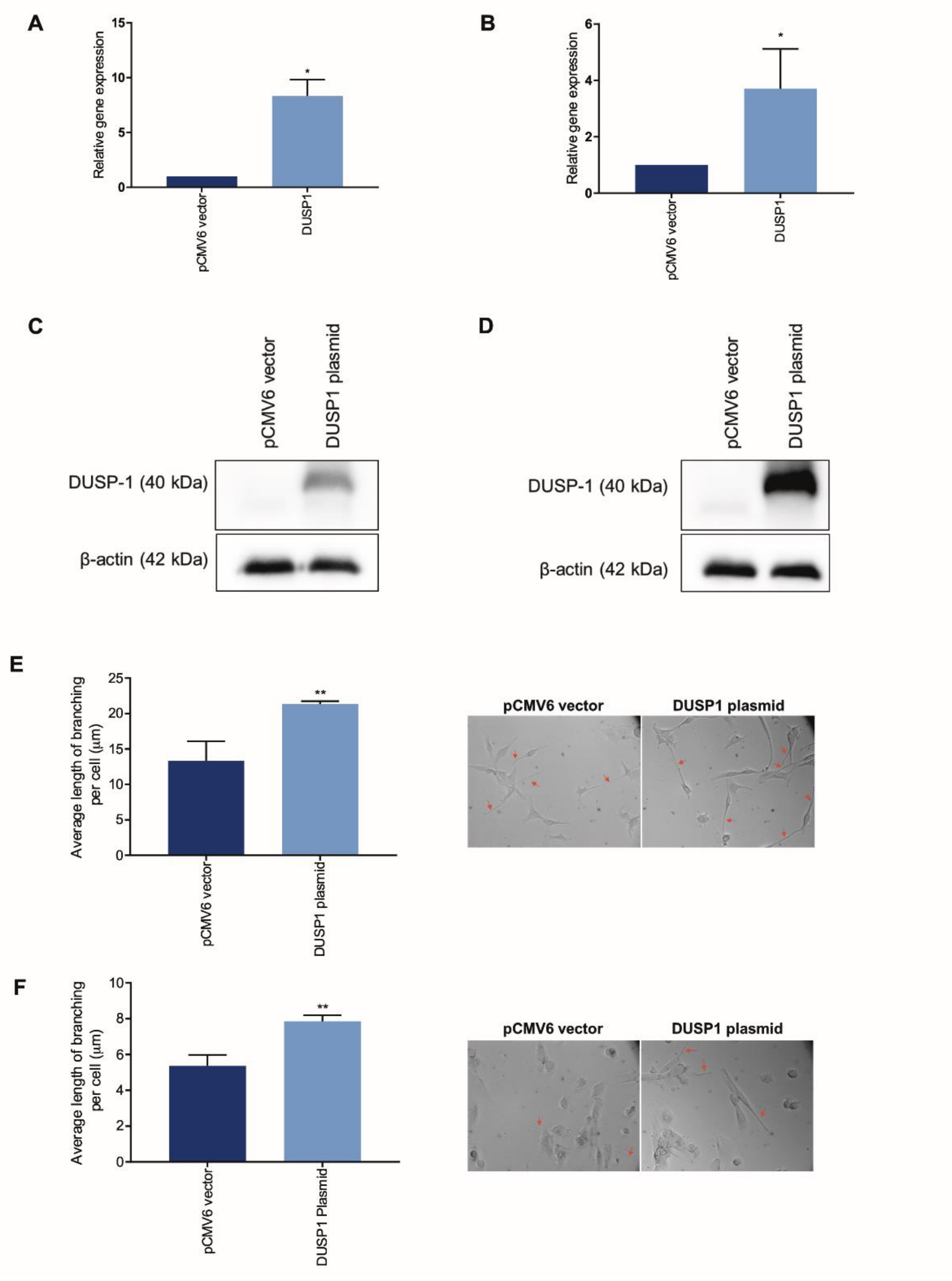


**Supplementary Figure 5: DUSP1 overexpression enhances neurite-like process outgrowth in U87 GBM cells and G179 GSCs. A-B** qRT-PCR was performed measuring relative mRNA expression of DUSP1 normalised to GAPDH after U87 GBM cells (A) and G179 GSCs (B) were treated with 1ng/µl DUSP1 overexpression plasmid for 48h (fold change compared to control levels 1). **C-D** Western blotting analysis of DUSP1 in U87 GBM cells (C) and G179 GSCs (D) treated with 1ng/µl DUSP1 plasmid for 48h normalised to loading control (β-actin) and CTRL. **E-F** Average length of neurite-like process was determined in U87 GBM cells (E) and G179 GSCs (F) treated for 48h with 1ng/µl DUSP1 plasmid compared with CTRL plasmid. Representative images are shown with neurite-like processes indicated with red arrows. The mean ± SD of minimum n=3 independent experiments is shown *p≤0.05 **p≤0.01 ***p≤0.001 (two-tailed t-test).
